# Business trends & challenges in Islamic FinTech: A systematic literature review

**DOI:** 10.12688/f1000research.109400.1

**Published:** 2022-03-18

**Authors:** Hatim Dawood, Dr. Fatin Al Zadjali, Mohammed Al Rawahi, Dr. Sitara Karim, Dr Mohamed Hazik

**Affiliations:** 1 Faculty of Business and Management, UCSI University, Kuala Lumpur, 56000, Malaysia; 2 College of Banking and Financial Studies, Muscat, Muscat, 112 Ruwi, Oman; 3 Department of Business Administration, Faculty of Management Sciences, ILMA University, Karachi, 75190, Pakistan; 4 Managing Director, Stellar Consulting Group, Singapore, 30009, Singapore

**Keywords:** Business trends, challenges, Islamic FinTech, systematic review, i-FinTech

## Abstract

**Background:** This systematic literature review (SLR) study is on Islamic financial technology (FinTech) business trends and challenges. It follows the Preferred Reporting Items for Systematic Reviews and Meta-Analyses (PRISMA) checklist. This research identifies the gaps in Islamic FinTech, which require further studies. Moreover, it highlights the issues raised during the coronavirus disease 2019 (COVID-19) pandemic.

**Methods:** This study is based on the FinTech business model (BM) classifications by Lee & Shin and Imerman & Fabozzi. Furthermore, the set of challenges used in this study are adopted from research by Lee & Shin and Li & Xu. The Scopus database was used to collect data using nine keywords. Articles and review papers published between 2016 and 2022 were included. Studies that were not published in English, and those with no ranking journals were excluded. The results were presented using bibliometric analysis.

**Results:** The results showed 36 articles discussing Islamic FinTech business trends and challenges, and most of these studies are conducted on FinTech crowdfunding vertical BM. By contrast, the most dominant horizontal BMs are FinTech regulation and FinTech funding BMs. The top challenge found in this study is the regulation management challenges. Moreover, there are remarkable dominating articles and reviews published in 2020 and 2021 discussing COVID-19.

**Conclusions:** This study concluded that many horizontal BMs were not covered in Islamic FinTech, especially horizontal technology BMs. Investment, property and insurance BMs are examples of unavailable articles. Islamic FinTech is considered a promising field due to the size of the opportunities it presents, the available capital, and the great demand for banking and financial products that comply with the Sharia. This study will help the Islamic FinTech industry grow and predict the demand, and provide an alternative to conventional banking FinTech and further boost the technology progress in the financial industry.

## Introduction

Financial technology (FinTech) is a new trend in the financial and banking industry, especially Islamic finance. This financial technology can promote Sharia-compliant products and increase financial inclusion among Muslim consumers and investors. Islamic finance is expected to play a vital and essential role after the coronavirus disease 2019 (COVID-19) pandemic ( Rabbani *et al*., 2021a). Thus, financial technology can help Islamic banks and financial institutions and enable them to compete with conventional banks and give them more decisive impetus. Islamic financial institutions can take advantage of fintech innovations to gain a competitive advantage, lower the prices of their products, and close the credit gap. Moreover, Islamic Fintech innovations can satisfy the younger (Z-generation) Muslim preferences ( Todorof, 2018). According to Todorof (2018), the most suitable Sharia-compliant FinTech innovations are credit platforms such as peer-to-peer (PTP) lending and automated investing.

**Figure 1.**
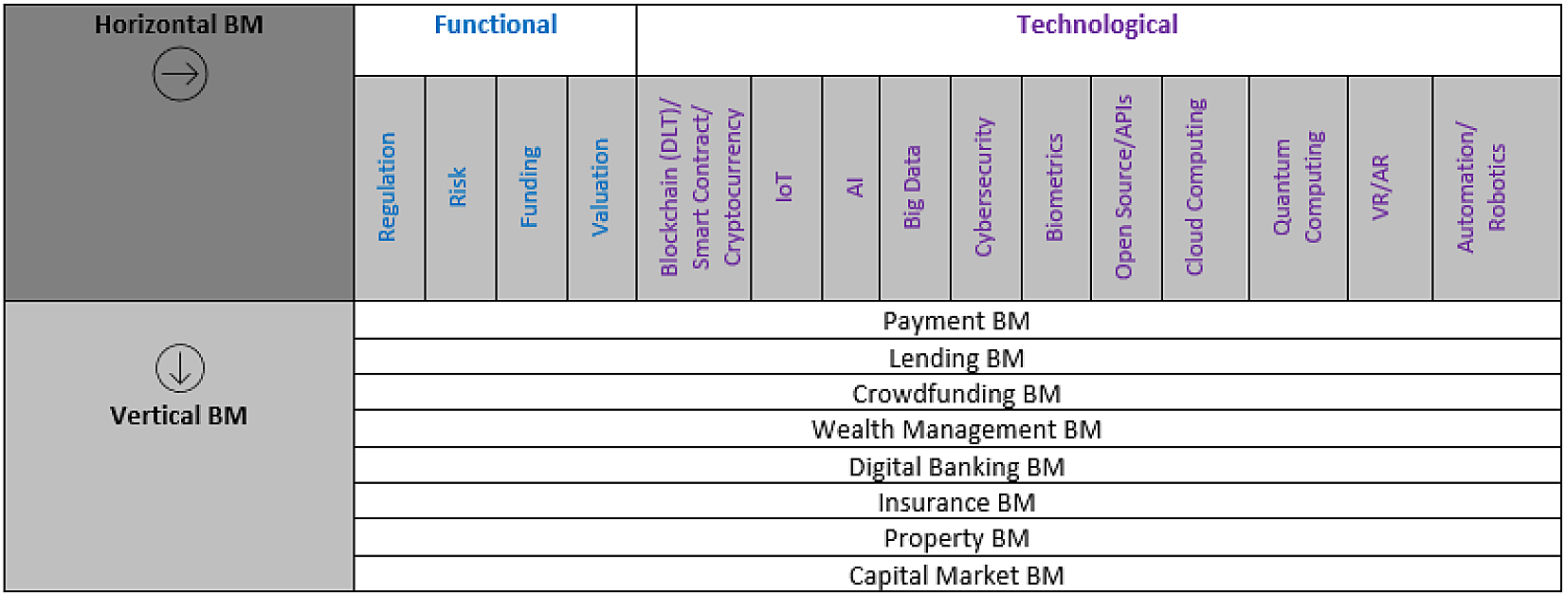
FinTech BMs (verticals and horizontals). FinTech (financial technology); BM (Business model); DLT (Distributed ledger technology); IoT (Internet of things); AI (Artificial intelligence); API (Applications interfaces); VR (virtual reality); AR (augmented reality).

The business model is one of the crucial elements in the FinTech ecosystem. Scholars categorized the FinTech business models into two types: vertical business model (FinTech verticals) based on the areas of the financial services ( Lee & Shin, 2018; Imerman & Fabozzi, 2020), and horizontal business model (FinTech horizontals) based on functional areas and emerging technologies ( Imerman & Fabozzi, 2020). The FinTech verticals are payments, wealth management, crowdfunding, lending, insurance, capital market ( Lee & Shin, 2018; Imerman & Fabozzi, 2020), digital banking and property business models ( Imerman & Fabozzi, 2020). By contrast, the FinTech horizontals have two more subcategories: functional subtype and technology subtype ( Imerman & Fabozzi, 2020). Financial regulations, risk management, funding, and valuation are functional subtypes. The technology subtypes are blockchain, internet of things (IoT), artificial intelligence, big data analytics, cybersecurity, biometrics, open application programming interfaces (APIs), cloud computing, quantum computing, virtual or augmented reality and automation or robotics ( Figure 1).

**Figure 2.**
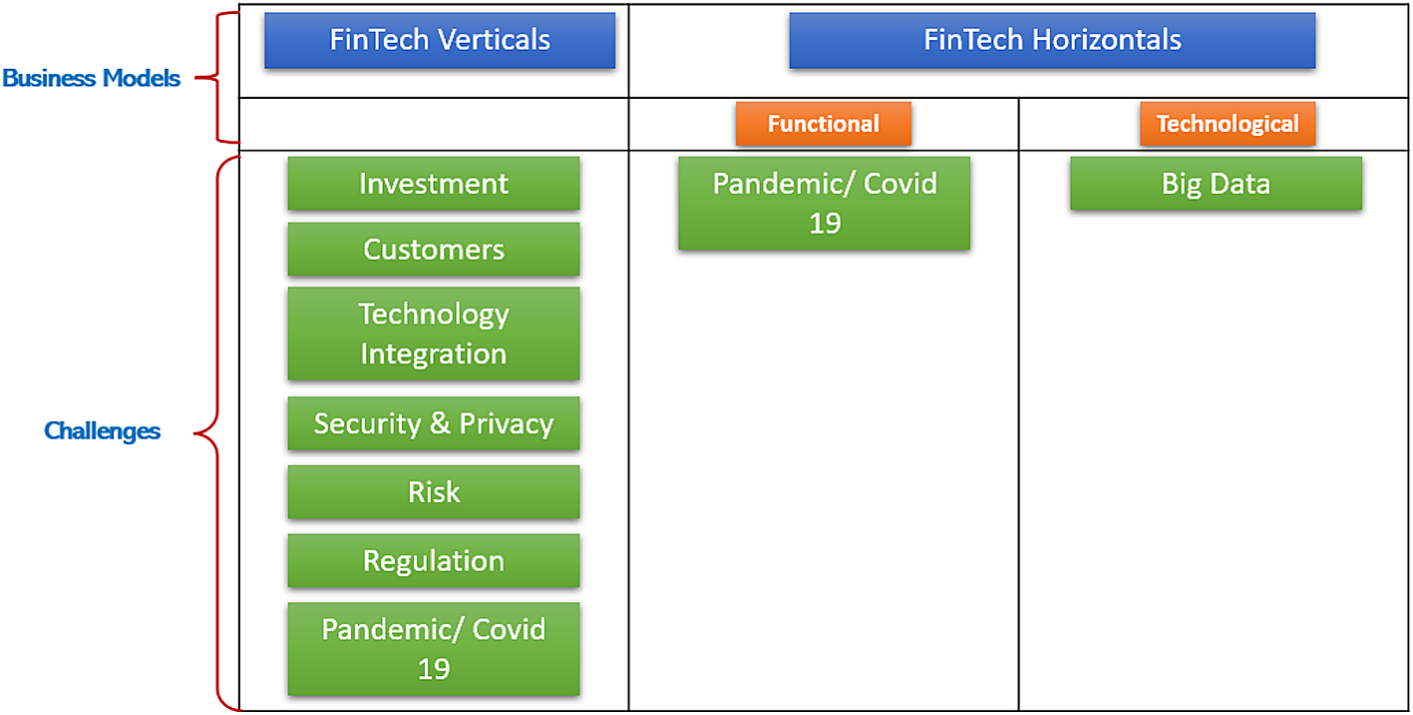
FinTech challenges to business models. Figure was produced by us based on our standing and conclusions of the source articles: ( Lee & Shin, 2018; Li & Xu, 2021). FinTech (financial technology); COVID-19 (coronavirus disease 2019).

Despite the benefits of financial technology to the financial industry, there are many challenges and obstacles that this technology encounters in the financial and banking sector. Lee and Shin (2018) defined six challenges facing the FinTech verticals business models: investment management, customer management, regulation, technology integration, security and privacy, and risk management. By contrast, Li and Xu (2021) added two more challenges: the impact of big data on FinTech horizontal-technologies business models and the impact of COVID-19 on FinTech verticals business model and FinTech horizontals-functional business models ( Figure 2).

The purpose of this research was to explore the Islamic FinTech business trends, challenges, and issues between 2016 and 2022, based on the Scopus database, using a systematic literature review (SLR) and bibliometric analysis.

We identified the need for a SLR in Islamic FinTech publications. Our objectives were to address the top trends and challenges in the Islamic FinTech context to provide a clear classification view of produced research articles and review articles and then highlight the current collaboration among the authors, countries and institutes worldwide to identify the gaps in the literature review of the Islamic FinTech.

In order to achieve the research purpose, we proposed four questions:

1. What are the business models trends in the Islamic FinTech publications?
2. What are the challenges and issues in the Islamic FinTech business models context?
3. What is the current collaboration among authors, countries and institutes in the Islamic FinTech business models?
4. What are the top issues that have been discussed about the impact of COVID-19 on Islamic FinTech?

## Background

### Islamic finance

Islamic finance is based on the quality and improvement of open customers' lives, equitable distribution of income, and social justice for members of society ( Rabbani *et al*., 2021a; Shaikh *et al*., 2020). They differ from other banks (financing) in that they apply Islamic laws (Shariah) to their products, services, transactions, and how they are presented to customers. For example, Islamic banks strictly avoid betting and waging (alqimar ^1^), gambling or speculation or unearned income (maysir ^2^), and excessive risk or uncertainty or deceit (gharar ^3^). Furthermore, the Shariah law prohibits alcohol, pork, and illegal drugs trading. Also, interest (riba ^4^) on deposits are not provided in Islamic banks, and typically, the Islamic banks invest in the desired assets by a customer through sharing the risk and profit derived from transactions. Thus, the bank buys the asset or product at a specific price and then sells it back to a customer at a higher price (musharakah ^5^) ( Todorof, 2018; Shaikh *et al*., 2020; Rabbani *et al*., 2020).

Moreover, in some cases, a bank owns the asset or product outright, and the customer receives ownership only once the payment is completely done, and hence the asset is owned by the partnership (murabaha ^6^). Islamic banks have existed in the market for more than thirty years. In Egypt, the first Islamic bank, Mit ghamr Savings Bank, opened in 1963 ( Todorof, 2018). However, their adoption is relatively lower than conventional banks. Malaysia, United Arab Emirates (UAE), Bahrain, the Kingdom of Saudi Arabia (KSA) and Oman are top pioneer worldwide leaders in Islamic banks ( Shaikh *et al*., 2020).

Four main factors led to the growth of Islamic banks: first, the Muslim population, which is 1.8 billion (2017) and is expected to reach 3 billion in the year 2060. Second, the youth of the Islamic world is distinguished by its young age, as the average age of youth in the Islamic world is 24 years, while the average age of young people in the world is 32 years, and therefore the youth of the Islamic world are more energetic, and inclined to technology, according to 2015 reports. Third, the volume of investment by the Islamic banks in the Islamic economy (Islamic countries) amounted to 745 million dollars between 2015 and 2018. Fourth, there is significant trade in Islamic economy lifestyle products, imports at $271.8 billion, and exports at $210.5 billion (2017) ^7^.

Islamic finance economy is growing and currently is worth $4.7 trillion. In UAE, the Islamic economy is contributing 10% of its GDP. Islamic finance assets are expected to achieve $3472 billion by 2024 ( Rabbani *et al*., 2021a). In 2021, the Islamic banks in Oman financed 4.7 billion Omani riyals, achieving a 12.9% increase compared to 2019. By contrast, the central bank of Oman (CBO) reported 4.3 billion Omani riyals as total deposits from Islamic banks and windows at the same period. In addition, Central Bank of Oman (CBO) announced that the total assets of Islamic banks and windows reached 5.8 billion Omani riyals in 2021 ^8^.

### FinTech

FinTech innovations have many contributions to the financial system, such as cost reductions, higher quality services, and increasing customer engagement and satisfaction. It helps firms gain competitive advantages and enhance efficiency ( Kou, Olgu Akdeniz, Dinçer, & Yüksel, 2021; Zouari & Abdelhedi, 2021). FinTech innovations use mobile technology to deliver banking services to customers as a complete business model. It uses high technologies such as blockchain, artificial intelligence and machine language, big data, and cloud computing to achieve its objectives.

According to Todorof (2018), more than 90% of the banks are expected to develop and implement mobile applications (m-bank). The bank service providers aim to enhance their customer's satisfaction and engage them via mobile. FinTech investment reached $93 billion in 2021 ^9^. The banks used mobile technology to provide various services for their customers, such as payment and remittance, lending and borrowing, investment, and insurance.

### FinTech business model trends

*FinTech payment business model (vertical)*

The FinTech payment business model (verticals) is divided into two market segments: consumer and retail, and wholesales and corporate payment. The consumer uses the payment frequently and daily, including mobile wallets, PTP mobile payments, foreign exchange and remittances, real-time payments such as utility bills, and digital currency solutions. These FinTech innovations provide consumers with convenience, speed of transactions and multi-channel accessibility ( Lee & Shin, 2018). According to Li and Xu (2021), the payment business model is the most mature among other FinTech business models. Most FinTech unicorns are from payment platforms such as PayPal, grab and Gusto ^10^.

*FinTech wealth management business model (vertical)*

The FinTech wealth management business model (verticals) automates wealth managers (Robo-advisors) to provide financial and investment consultation. It uses a specific set of machine learning algorithms to advise the investors based on their preferences and characteristics ( Lee & Shin, 2018). Li and Xu (2021) expected that the coming years would be crucial for this type of FinTech business model for two reasons: first, the FinTech retail solutions are growing for the Z-generation who are swift adopters of FinTech products. Second, pandemics such as COVID-19 boost the attraction of this type of business model as the customers were limited in different financial and banking sectors.

*FinTech crowdfunding business model (vertical)*

The FinTech crowdfunding business model (verticals) is based on a group of people or corporates gathered to create funds. Li and Xu (2021) clearly defined FinTech lending business models such as PTP and FinTech crowdfunding business models. They said lending platforms enable borrowers to obtain funds from others to invest, while crowdfunding platforms allow the borrowers to obtain funds by giving the lenders (or investors) a stake in the project's success. Typically, the FinTech crowdfunding business model has three stakeholders: the fund contributors (investors or lenders), the customer or project (borrowers) and the FinTech platform that facilitate the engagement between all the stakeholders. The most common FinTech crowdfunding business models, according to Lee and Shin (2018), are reward-based, donation-based, and equity-based. Reward-based crowdfunding is formed for small business and innovation projects. In a reward-based crowdfunding business model, the borrowers decide the interest rate and can choose any guarantee to refund within a stipulated period. The borrowers usually give some reward to the lenders. Donation-based crowdfunding focuses on raising funds for charity projects from donors who receive nothing in return. Equity-based crowdfunding is also designed for small and medium companies (SMEs), allowing entrepreneurs to reach investors interested in acquiring equity in their startups. The SME shares ownership percentage with the investors (lenders) ( Lee & Shin, 2018).

*FinTech lending business model (vertical)*

The FinTech lending business models (verticals) such as PTP, agency lending, and invoice financing are significant trends. It allows individuals or financial institutions to lend to other individuals or SMEs. Typically, this type of FinTech business model offers low-interest rates and provides the fund in less time ( Lee & Shin, 2018; Reiners, 2018).

*FinTech capital market business model (vertical)*

The FinTech capital market business model (verticals) is about trading that allows investors and traders to connect, share and place a buy or sell order of stocks and goods and monitor real-time risks. Foreign currency transactions are another example of the FinTech capital market business model that reduces the barriers and costs for individuals and firms to engage in foreign currency transactions using mobile technology ( Lee & Shin, 2018).

*FinTech insurance (InsurTech) business model (vertical)*

The FinTech insurance services (InsurTech) business model (verticals) enables a direct relationship between the insurer and the customers to meet customers' needs and offer them insurance products ( Lee & Shin, 2018). According to Li and Xu (2021), insurance companies depend on advanced data analytics more than ever. They added that insurance companies have more access to real-time and near-continuous data about various aspects such as health, driving habits, and other safe and healthier behaviors, allowing them to restructure insurance policies and prices dynamically. InsurTech was expected to be the biggest FinTech vertical business model to observe going forward ( Li & Xu, 2021).

*FinTech digital banking (neo-banking) business model (vertical)*

The FinTech digital banking business model (verticals) includes online and mobile banking operations. Neo-banks or challenger-banks that provide all banking services through digital banking with little or no physical presence are becoming more widely known ( Li & Xu, 2021). The complete dependence on technology allows these digital banking firms to obtain competitive advantages. Pandemics and other financial crises lead to reduced customer trust in big banks. Furthermore, global banks encourage their customers to use neo-banking services in response to COVID-19. The rise of neo-banking can lead to three consequences, according to Li and Xu (2021): first, shortages of funds. Second, acceleration of the partnership between FinTech startups in the banking industry and the conventional banks. Third, customers are frustrated from conventional banks' services compared to the neo-banks services “FinTech startups in the banking industry” because the neo-banking are superior, user-friendly, easy to use, and more valuable. Thus, as a result, more money is earned by the neo-banking.

*FinTech property (PropTech) business model (vertical)*

The FinTech PropTech business model (verticals) is digitalizing the various activities in real estate and shifting its services to more user-friendly using technologies. It allows stakeholders to make acquisition and disposal decisions and manage a portfolio of real estate properties. Furthermore, it enables buyers to visit properties and provide properties data virtually. Moreover, it allows for the crowdfunding of real estate projects ( Li & Xu, 2021).

*FinTech regulation business model (functional horizontals)*

According to Li and Xu (2021), financial regulation is the most impacted area because of FinTech innovations. The increasing reliance on technology and economies of scale in information technology applications leads to many risks on consumers, firms, and financial stability and hence the regulator responds to each type of risk differently. For instance, regulators respond to consumers' risks by setting policies that protect consumer information and privacy. Furthermore, regulators respond to firms' risks by imposing governance, risk management, and operational resilience. KPMG (2019) listed these types of responses that vary based on the impact area.

Moreover, regulators respond to financial stability risks by data and information gathering and regulatory interventions. Technological innovations in financial regulation are called regulatory technology (RegTech) or supervisory technology (SupTech). These technologies usually ensure the routine tasks of compliance are intelligent and efficient using data analytics and automation.

*FinTech risk management business model (functional horizontals)*

The impact of FinTech innovations on bank risk-taking is heterogeneous ( Deng, Lv, Liu, & Zhao, 2021). Typically, FinTech innovation can impose certain risks to consumers, firms, and regulators. Lack of awareness, financial exclusion and personal or financial information leakage are risks to consumers. By contrast, firms can be impacted by different types of risks due to FinTech innovations such as governance, technology, operational, data management, money laundering, legal, and many more risks. On the other hand, regulators and central banks can be impacted by FinTech innovations that can cause higher risks to financial stability, such as uncontrolled alternative channels of financial intermediation, crypto-assets, and their vulnerabilities, and many more.

*FinTech funding business model (functional horizontals)*

Banks play a vital role in financing FinTech startups. The FinTech funding business model is about funding FinTech trends by digital entrepreneurs “investors or venture capitalists”. It operates in the retail banking and SME banking spaces to target the underbanked population in emerging markets to enhance financial inclusion ( Pranay & Mandy, 2019; Bican & Brem, 2020). However, banks have changed their strategy and role from traditional money lending (debt funding) and becoming a stakeholder in FinTech (equity investors) due to innovations in digital technology ( Hommel & Bican, 2020).

*FinTech valuation business model (functional horizontals)*

The FinTech valuation business model is about promising startups and seasoned firms. This valuation investigates if FinTech follows the typical evaluation patterns of financial intermediaries or technological firms. The evaluation patterns usually used discounted cash flows (DCF) or comparable market metrics ( Visconti, 2020).

*FinTech blockchain business model (technology-horizontals)*

The FinTech blockchain business model is one of the most common research topics in the FinTech context ( Li & Xu, 2021). It has many applications in the financial industry, such as cryptocurrencies, clearing and settlement in over-the-counter derivative markets, insurance, trade finance, real estate, and smart contract ( Imerman & Fabozzi, 2020). Blockchain technology helped conventional banking services with better transaction security and faster money exchanges at lower costs locally and internationally ( Lee & Shin, 2018). Furthermore, blockchain technology helps FinTech startups obtain competitive advantages, become more transparent to customers, have a broader reach, and be more efficient than their conventional counterparts ( Rabbani *et al*., 2021a).

*FinTech internet of things (IoT) business model (technology-horizontals)*

The FinTech IoT is about multiple devices such as smartwatches, fitness trackers, smart home devices, smart thermostats, and many others, which are connected, and their data flow is used in financial applications. This type of FinTech can help the consumer reduce the cost of living and help the supplier collect the payments more efficiently ( Imerman & Fabozzi, 2020). Combining IoT and artificial intelligence lead to sustainable finance ( Pustokhina, Pustokhin, Mohanty, García, & García-Díaz, 2021).

*FinTech artificial intelligence (AI) business model (technology-horizontals)*

According to Imerman and Fabozzi (2020), much of the digital transformation that we see in financial services can be attributed to automation and the integration of AI. In addition, AI is used with big data analytics to improve business intelligence. Furthermore, AI can treat complex code-based problems, assisting humans in decision-making. Moreover, AI can reduce cost, assist regulatory and policymaking, reduce operating errors and enhance productivity ( Rabbani *et al*., 2021a).

*FinTech big data analytics business model (technology-horizontals)*

The FinTech big data business model involves a complex process of examining large data sets to uncover information, pattern, unknown correlations, market trends and customer preferences and thus help in decision making ( Visconti, 2020). Imerman and Fabozzi (2020) attributed the reason for the development of the FinTech industry to the power of big data analytics on unstructured data and obtain more valuable insights into various aspects of FinTech verticals business models such as lending, crowdfunding, and many more. Furthermore, big data analytics can reduce costs and enhance the ability to conduct any actions in real-time ( Hommel & Bican, 2020). Moreover, big data can be further used to achieve customer expansion, risk evaluation and credit decision-making ( Deng, Lv, Liu, & Zhao, 2021).

*FinTech cybersecurity business model (technology-horizontals)*

The FinTech cybersecurity business model is about the financial and personal data breaches from banks and other intermediaries such as FinTech platforms (FinTech startup) ( Imerman & Fabozzi, 2020). Therefore, a series of anti-fraud initiatives shall be considered to prevent and mitigate data breaches and enhance data security. Antifraud can be done using tools, procedures, and techniques to increase customer trust.

*FinTech biometric business model (technology-horizontals)*

Biometric technologies are used widely in mobile devices to enhance security and data privacy ( Wang, 2021). The FinTech biometric business model uses and implements specific mechanisms to authorize customers to access data ( Imerman & Fabozzi, 2020). It enables the right person to access the correct data. Furthermore, it utilizes customer physical characteristics such as fingerprint, blood vessels in the retina, facial structure, and others to authorize the customer to access a specific set of data digitally.

Recently, biometric technologies allowed the customer to pay digitally or perform financial transactions using his or her physical characteristics ( Imerman & Fabozzi, 2020). This helps customer to perform any transaction easier and in a convenient manner. Furthermore, it is ideal for collecting rich data obtained using sensors and AI in mobile devices ( Wang, 2021). Wang (2021) said that biometrics could enhance FinTech platforms' security, protect customers' data, and enhance their trust because it can reduce their financial and personal information leakage.

*FinTech open APIs business model (technology-horizontals)*

The FinTech open APIs business model uses application programming interfaces in FinTech platforms. Open APIs/banking provides a gateway for FinTech innovations to create value offerings through data. It provides rich data ( Imerman & Fabozzi, 2020) and boosts FinTech innovations using mobile technologies. Furthermore, it has four benefits: increased customer-centricity, improved operational efficiency, generating higher profitability and increased interoperability ^11^.

*FinTech cloud computing business model (technology-horizontals)*

The FinTech cloud computing business model migrates local/on-site storage to the internet using virtual servers. Therefore, the entire firm can access the same information, same format and from anywhere ( Imerman & Fabozzi, 2020).

*FinTech quantum computing business model (technology-horizontals)*

FinTech quantum mechanics develop processors to calculate complex equations or transactions more quickly than traditional computers ( Imerman & Fabozzi, 2020).

*FinTech virtual/augmented reality business model (technology-horizontals)*

Virtual reality (VR) and related technologies create virtual environments with key characteristics representing real-world situations. The primary motivation for using VR in financial trading is to enhance the user perception and cognition about the complexity of the financial market mechanisms and manifestations. The added value of VR modelling the financial market is its advanced visualization technique and metaphoric representation that mimic real-world situations.

*FinTech robotics/automation business model (technology-horizontals)*

The FinTech robotic business model is highly integrated with artificial intelligence in the financial industry. According to Belanche *et al*. (2019), there is a high demand for robotic and AI systems to interact with humans. However, differences in the individual's familiarity with robotic systems play a significant role in adopting Robo-advisors ( Belanche, Casaló, & Flavián, 2019).

### Challenges of FinTech business models

Fernandez-Vazquez *et al*. (2019) found in their studies about the blockchain in FinTech context that the results show a deep focus on challenges such as security, scalability, legal and regulatory, privacy or latency, with proposed solutions still far from being effective.

*The investment management challenge*

The FinTech investment management challenges are (1) the ability to evaluate and select a project without a FinTech projects portfolio that can deliver the most competitive and profitable outcomes, and (2) the response of financial institutions to FinTech startups whether to compete or collaborate ( Lee & Shin, 2018).

*The customer management challenge*

FinTech customer management is about acquiring and retaining customers. Customer's retention in FinTech innovation is achieved through different strategies such as being responsive, easy to access, convenient, and real-time customer support using AI and big data analytics. There are multiple FinTech services and multiple channels used by different customers. Nevertheless, human interaction is still required to enhance customer support ( Lee & Shin, 2018).

*The regulation management challenge*

The FinTech regulation challenges are capital requirements, anti-money laundering and privacy and security concerns. There are specific rules and regulations for FinTech innovations, such as the lending business model ( Lee & Shin, 2018).

*The technology integration challenge*

FinTech technology integration is essential for providing a complete service to a customer. Technology integration challenges reside in the existing legacy systems ( Lee & Shin, 2018).

*Security and privacy challenges*

The FinTech security and privacy challenges include cybersecurity and customer information being stored in a secure place. Data loss, mobile loss, or cyberattack can be a possible threat to FinTech innovation. The payment or e-wallet transactions through FinTech innovation must be secured via mobile technology or web-based technology. Trust in FinTech mobile technologies can be increased when customers attitudes change positively toward the security of transactions and information privacy ( Lee & Shin, 2018).

*The risk management challenge*

FinTech risk management challenges include many types of risks such as financial, liquidity, interest rate ( Lee & Shin, 2018), operation, legal, and security and privacy risks ( Ryu & Ko, 2020). For instance, the lending business model allows borrowers with high credit risk to obtain loans or funds due to the difference in credit risk assessments used in traditional financial institutions ( Lenz, 2016; Ozili, 2018).

*The impact of big data challenge*

According to Li and Xu (2021), big data has a significant effect on the market and has a crucial role in the digital economy. It is the best technological tool to be used for FinTech innovations. It can be integrated with other technological tools such as cloud computing, AI and IoT, leading to massive growth in data. Therefore, data security and its usage efficiency played a vital role in FinTech innovation.

*The impact of COVID-19 challenge*

Li and Xu (2021) said that FinTech innovations faced many unpredictable risks because many financial institutions offered online services during the COVID-19 pandemic. For example, COVID-19 affected the FinTech lending business model ( Najaf, Subramaniam, & Atayah, 2021) and changed its fundamental determinants.

## Methods

**Figure 3.**
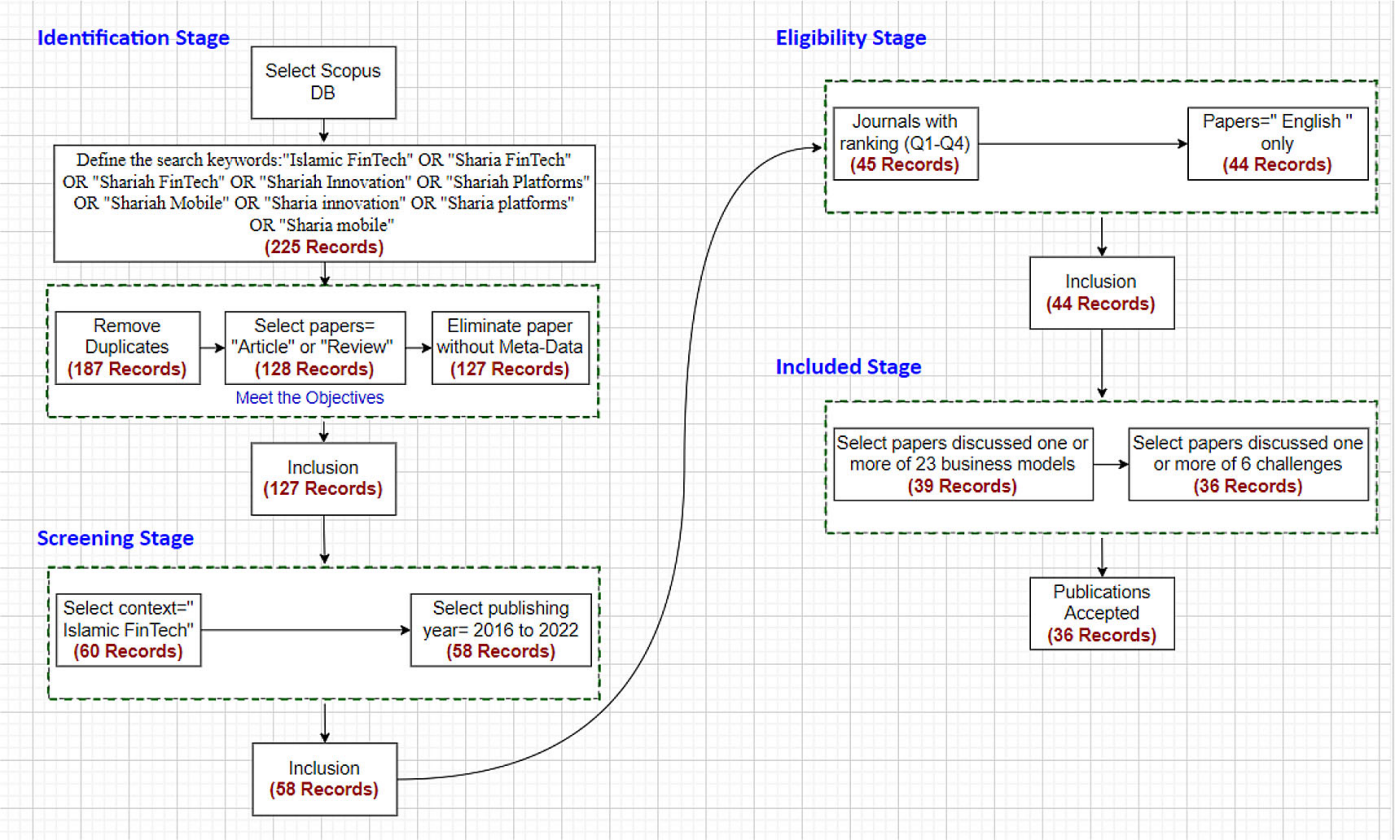
A systematic literature review. FinTech (financial technology); DB (Database).

This study adopts a SLR using the Preferred Reporting Items for Systematic Reviews and Meta-Analyses (PRISMA) guidelines ( Dawood *et al*., 2022) to provide a transparent, reproducible, and scientific literature review ( Casino, Dasaklis, & Patsakis, 2019) of Islamic FinTech. The SLR protocol was developed to analyze quantitative and qualitative data using meta-analysis to explore the Islamic FinTech business trends and challenges in the selected studies from the Scopus database ( Nasir *et al*., 2021). Furthermore, this research used bibliometric analysis to explore recent collaborations among scholars in the Islamic FinTech domain and address current issues. The SLR protocol's methodological approach includes four stages: the identification stage, the screening stage, the eligibility stage, and the including or data extracting stage ( Figure 3).

### Identification stage

The research questions for this study were constructed based on the need to identify the gaps and trends in the Islamic FinTech domain. The Scopus database ^12^ was used to extract the studies as the Scopus database is widely considered by the scholars for SLR to confirm the appropriateness and quality of the studies included in the study. The keywords used to find the data from the Scopus database were classified according to Islamic finance and technology context strategy. The keywords selected for data extraction from the Scopus database were: Islamic FinTech, Sharia FinTech, Shariah FinTech, Shariah Innovation, Shariah Platforms, Shariah Mobile, Sharia innovation, Sharia platforms, and Sharia mobile. Each keyword was used to search matched articles in the Scopus database.

• Population: as per nine keywords.
• Outcomes: 225 records (papers).

The initial outcomes included duplication due to multiple keywords used in the search process. Duplicated records were removed.

The outcome included all types of papers such as conference papers, books, reviews, articles and many more. Therefore, this study only selected articles and review papers because they are usually written by researchers or scientists and published in a scholarly, peer-reviewed journal ^13^. We found one article with no meta-data (authors and other information are missing). Research articles are based on primary data and the most common type of journal article. They present and produce new knowledge. By contrast, review articles provide constructive analysis on secondary data to identify gaps or problems and provide future recommendations for further studies ^14^.

### Screening stage

We narrowed the outcome from the identification stage by screening all articles and searching for articles written in the Islamic FinTech context. Articles published in Islamic FinTech published between 2016 and 2022 were selected.

### Eligibility stage

In this stage, we accepted only articles published in journals with rank either Q1 or Q2 or Q3 or Q4 according to SCIMAGO institutions rankings ^15^. Non-ranked journal's articles or non-English language articles were removed at this stage.

### Data extraction stage

The data extraction stage, also referred to as including stage, is the final stage for accepting articles. All selected files were imported in NVIVO software (NVivo, RRID:SCR_014802) ^16^. Then using search options, we searched for 23 types of FinTech business models, both vertical and horizonal, as per the classifications by Lee and Shin (2018) and Omerman & Fabozzi (2020). Using codes, we coded each type of the eight challenges in FinTech as classified by Lee and Shin (2018) and Li and Xu (2021). Any challenge found related to one article that discuss multiple FinTech business model, is classified under mix of business model category. However, we found that some articles discussed one or more challenges of the eight challenges but there are other business models that are not classified from the 23 types of business models based in this study, thus these were kept under the “Other” category. We read each article and categorize each issue under of these eight challenges. This process was carried out by three authors, and then two supervisors. The NVIVO software (free trial) gave a list of issues under each coded challenge. We filtered and combined the issues that were identical but different phrases.

### Synthesis methods

To prepare the Research Information Systems (RIS) (Research Information Systems *.ris) file needed for the VOSviewer (version 1.6.17), Mendeley (version 1.19.8) software (Mendeley Data, RRID:SCR_002750) was used to import all articles (36 data files). All files were imported to Mendeley, and by using screening the list, we found articles with no journal details and by using the URL link provided in the Excel file (Microsoft Excel, RRID:SCR_016137), were able to capture all data required to make the document organized, such as title names, author names, years, journal names, International Standard Serial Numbers (ISSNs) and DOIs, *etc.*

Furthermore, meta-analysis was used to extract the challenges and issues from the selected articles using NVIVO (free trial) software.

Then, we created a map based on bibliographic data. This was to create a co-author, keyword co-occurrence, citation, bibliographic coupling, or citation map based on bibliographic data. The data were taken from the RIS file generated from Mendeley.

### Data analysis

The data analysis consists of three types of analysis: meta-analysis (NVIVO, free trial), bibliometric analysis (VOSviewer, 1.6.17), and other analysis (MS-Excel 360). Meta-analysis was used to find out the challenges and issues of the articles. Bibliometric analysis was used to find the following information: co-occurrence network of authors keywords, collaboration network of countries, collaboration network of authors, and collaboration network of institutes. We called the final type of data analysis ‘other analysis’ and it covered the top journals for the selected articles.

## Results

PRISMA identification stage yield a total of 225 articles and review papers were matched by the SLR protocol and analyzed for this study. Removal of the duplicated records yielded an outcome of 187 records. Choosing articles and review articles resulted in 128 studies, eight review articles, and 120 research articles. We found one article with no meta-data (authors and other information are missing). As a result of the identification stage, 127 records (papers) were selected.

In screening stage, we found that out of 127 articles, only 60 articles were published in the Islamic FinTech context. These articles were published between 2016 and 2022. Only two articles were published before 2016. Therefore, we selected those articles published between 2016 and 2022. The total number of articles was 58.

The eligibility stage yielded an outcome of 45 articles. Moreover, one article was removed because it was not in English; therefore, the total outcome was 44 articles accepted and were eligible to include in our research.

For data extraction (inclusion stage), we scanned 44 articles and selected only 39 papers discussing one of the business models Imerman and Fabozzi (2020) listed. We excluded around five articles related to the factors affecting the adoption and FinTech acceptance and did not discuss any business models. Finally, we scanned 39 articles to search for those articles that covered one or more of the six challenges mentioned by Lee and Shin (2018). We found that only 36 articles discussed these challenges. Therefore, our research concluded that 36 articles discussed and included at least one business model, vertical or horizontal or both, and at least one challenge from the six challenges.

The results section includes several types of analyses on the extracted data that satisfy the questions of this study. This section covers meta-analysis, bibliometric analysis, and other analyses.

### Meta-analysis of the articles selected

The meta-analysis used in this study was performed to explore the business model trends and challenges discussed in each article. Furthermore, it was used to identify the main issues of the selected articles based on a particular challenge.

*Islamic FinTech business model trends*

Table 1 shows the number of articles published in vertical and horizontal Islamic FinTech business models. The trends of the business models were obtained by reading and scanning each extracted article's title, abstract, introduction, discussion, and conclusion sections (total 36 articles). From a horizontal business models perspective, the regulation business model and funding business model are the top trending in the Islamic FinTech context and are discussed in seven articles. By contrast, the crowdfunding business model is the top trending among the other vertical business models. The crowdfunding vertical business model is discussed in twelve articles. Business models such as valuation, the internet of things, big data, cybersecurity, open-source, cloud computing, quantum computing, virtual reality, augmented reality, and robotics are still not discussed in the Islamic FinTech context.

**Table 1.** Total articles per Islamic FinTech BM.

Vertical BM	Horizontal BM																	Total
	*Functional*				*Technological*											*Mix of horizontals*	*Other*	
	Regulation	Risk	Funding	Valuation	Blockchain (DLT)/smart contract/Cryptocurrency	IoT	AI	Big data	Cybersecurity	Biometrics	Open-source APIs	Cloud computing	Quantum computing	VR/AR	Automation/Robotics			
***Payment BM***					1												1	**2**
***Lending BM***			1		1													**2**
***Crowdfunding BM***	1	1	6				1										3	**12**
***Wealth management BM***																		**0**
***Digital banking BM***																		**0**
***Insurance BM***																		**0**
***Property BM***																		**0**
***Capital market BM***	1				2												4	**7**
***Mix of verticals***	2				1												3	**6**
***Other***	3	2			1		1											**7**
**Total**	**7**	**3**	**7**	**0**	**6**	**0**	**2**	**0**	**0**	**0**	**0**	**0**	**0**	**0**	**0**	**0**	**11**	**36**

FinTech (financial technology); BM (Business model); DLT (Distributed ledger technology); IoT (Internet of things); AI (Artificial intelligence); API (Applications interfaces); VR (virtual reality); AR (augmented reality).

The horizontal-technological business models have not been paid much attention in articles about Islamic FinTech. Nevertheless, vertical business models such as lending, crowdfunding, payment, and a mix of other vertical business models were discussed independently of the horizontal business models.

*Islamic FinTech business model challenges*

NVivo software was used in this study to perform selective coding to classify all issues per challenge in the Islamic FinTech context and adapt it to the purpose of this study. Some articles discussed more than one challenge, and others discussed challenges not classified neither by Lee and Shin (2018) nor by Li and Xu (2021), which are the theoritical framework for this study and hence they categorised as Mix of business models or other.

The regulation management challenge is the top challenge discussed in the selected articles. It is discussed in seven articles, followed by the customer management challenge in six articles, and the COVID-19 pandemic challenge in five articles ( Table 2). The selected articles did not cover technology integration challenges, security and privacy challenges ( Lee & Shin, 2018), and big data impact challenges ( Li & Xu, 2021).

**Table 2.** Challenges per each Islamic FinTech BM.

Vertical BM	Horizontal BM																
	*Functional*				*Technological*											*Mix of horizontals*	*Other*
	Regulation	Risk	Funding	Valuation	Blockchain (DLT)/smart contract/Cryptocurrency	IoT	AI	Big data	Cybersecurity	Biometrics	Open-source APIs	Cloud computing	Quantum computing	VR/AR	Automation/Robotics		
***Payment BM***					Regulation Challenge												Customer Challenge
***Lending BM***			Customer Challenge		Multiple Challenge												
***Crowdfunding BM***	Regulation Challenge	Risk Challenge	• Investment Challenge • Multiple Challenge • Risk Challenge • Regulation Challenge • Covid-19 Challenge				Covid-19 Challenge										• Customer Challenge • Multiple Challenge • Covid-19 Challenge
***Wealth management BM***																	
***Digital banking BM***																	
***Insurance BM***																	
***Property BM***																	
***Capital market BM***	Multiple Challenge				Regulation Challenge												• Multiple Challenge • Regulation Challenge • Customer Challenge
***Mix of verticals***																	• Covid-19 Challenge • Customer Challenge
***Other***	• Regulation Challenge • Customer Challenge	• Multiple Challenge • Covid-19 Challenge			• Other Challenge • Regulation Challenge		Other Challenge										

FinTech (financial technology); BM (Business model); DLT (Distributed ledger technology); IoT (Internet of things); AI (Artificial intelligence); API (Applications interfaces); VR (virtual reality); AR (augmented reality).

There is a clear and vital trend in the challenges towards regulation issues, both for Islamic cryptocurrency and Shariah-compliant financial technology regulation. Furthermore, important issues are discussed in extending customer adoption and acceptance of Islamic FinTech. Islamic FinTech can provide solutions for issues that arised due to the COVID-19 pandemic and can contribute to overcoming this pandemic as it facilities unique services to individuals and small and medium companies have been raised. Table 3 shows the most common issues discussed in each type of Islamic FinTech challenge in the selected articles.

**Table 3.** Issues per Islamic FinTech challenge found in this study.

Challenge	Issues
(1) Regulation management challenge	1. Islamic Cryptocurrency and its regulations based on with Shariah law ( Abdeldayem, al Dulaimi, & al Dulaimi, 2021; Kirchner, 2020). 2. Engagement of Islamic FinTech in money creation ( Altwijry, Hassan, & Selim, 2021). 3. Sharing the Islamic Fintech ethical benefits ( Altwijry, Hassan, & Selim, 2021). 4. Government role in regulation of the Islamic FinTech based on Shariah law ( Hendratmi, Ryandono, & Sukmaningrum, 2019; Darmansyah, Fianto, Hendratmi, & Aziz, 2020). 5. Shariah compliance shapes the attitudes and intention to use Islamic FinTech innovations ( Hasnan, 2021). 6. Regulation of assets based and backed Murabaha sales ( Gundogdu, 2016). 7. Halal products and services which guarantee legitimacy of the transactions and conformant to Islamic business rules ( Jailani, Al-Aaidroos, Mukhtar, Abu Bakar, & Ismail, 2020). 8. A comprehensive legal framework for Islamic FinTech against financing terrorism, rampant occurrence of illegal FinTech businesses and consumer disputes in the FinTech sector ( Muryanto, Kharisma, & Ciptorukmi Nugraheni, 2021; Abdullah & Umar, 2017). 9. Introducing FinTech interest-free foreign exchange and its benefits to the Islamic Banks ( Selim, 2021). 10. The effects of eliminating riba (interest) using FinTech innovation compliant with Shariah law ( Selim, 2021). 11. The effectiveness of Islamic FinTech regulations ( Nastiti & Kasri, 2019). 12. The efficiency and effectiveness of Ṣukūk offerings ( Busari & Aminu, 2022).
(2) Customer management challenge	1. The influence of FinTech innovations on customer retention in Islamic banks ( Baber H., 2019a). 2. Young Muslim generations and cash-waqf transactions involvement ( Nour Aldeen, Ratih, & Sari Pertiwi, 2021). 3. Consumer trust in Islamic FinTech using integrity, ability, and benevolence ( Usman, Mulia, Chairy, & Widowati, 2020). 4. Factors affecting SMEs adoption of Islamic peer-to-peer lending platforms such as loan processes, interest rates, loan costs, loan amounts and loan flexibility ( Rosavina, Rahadi, Kitri, Nuraeni, & Mayangsari, 2019). 5. The impact of Shariah compliance information on customer satisfaction ( Baber, 2019b). 6. The impact of Halal knowledge on the individual intentions ( Berakon, Wibowo, Nurdany, & Aji, 2021). 7. The impact of FinTech embeddedness in financial transactions and demand and supply in terms of achieving cost efficiencies and extend outreach ( Shaikh S. A., 2021).
(3) Risk management challenge	1. Mudharabah platforms risks ( Ishak & Rahman, 2021). 2. Mitigating risks using Islamic FinTech to monitor projects, enhance regulatory related to project investment funds, enhance mudharabah practice and creating awareness about mudharabah philosophy ( Ishak & Rahman,2021; Ishak, Kamaruddin, & Aderemi, 2021). 3. Islamic banking stability and its role in risks mitigation ( Banna, Hassan, Ahmad, & Alam, 2021).
(4) Investment management challenge	1. Islamic crowdfunding platforms and raising capital for entrepreneurship ( Rahman, Mohd Thas Thaker, & Duasa, 2020). 2. The role of crowdfunding platforms in linking cross-geographical investors with SMEs ( Hendratmi *et al*., 2020). 3. Factors affecting the acceptance of Islamic crowdfunding by youth entrepreneurs ( Salim, Kassim, & Thaker, 2021). 4. Islamic commodity future contract derived from asset-backed Murabaha ( Gundogdu, 2016). 5. Shariah-compliant products migration to electronic trading platforms ( Gundogdu, 2016).
(5) COVID-19 impact challenge	1. The role of Islamic FinTech products to fight the impact of ongoing Corona virus (Covid 19) on the individuals and SMEs ( Syed, Khan, Rabbani, & Thalassinos, 2020; Rabbani *et al*., 2021a; Ascarya, 2021). 2. The impact of Covid-19 pandemic on the number of Islamic mobile banking transactions ( Berakon *et al*., 2021). 3. The new set of financial services, strategies, and technologies to overcome Covid-19 pandemic (M., Rabbani, Asad, & Ali, 2020). 4. The utilization of Islamic FinTech innovation to address Covid-19 pandemic challenge (M., Rabbani, Asad, & Ali, 2020).
(6) Other challenges	1. The link between Islamic FinTech and the Islamic banks performance ( Almulla & Aljughaiman, 2021). 2. Islamic FinTech sustainability ( Chong, 2021; Rabbani *et al*., 2021b; Banna, Hassan, Ahmad, & Alam, 2021; Abdullah & Umar, 2017). 3. The algorithmic protocol to validate smart contracts (smart Sukuk) ( Chong, 2021). 4. The collaborations between Islamic banks, FinTech players and start-ups ( Auwal Adam Sa’ad, Khaliq Ahmad, & Saleh, 2019).

FinTech (financial technology); COVID-19 (coronavirus disease 2019).

### Bibliometrics analysis

According to Li and Xu (2021), a bibliometric analysis leads to more comprehensive research and discloses distinctive statistical patterns. Bibliometrics can visualize results using science mapping analysis tools such as VOSviewer software; thereby, the analyzed results become more readable and more precise. The bibliometric analysis is used in different research contexts such as blockchain, cryptocurrency, financial innovation, COVID-19 and many more. This study used VOSviewer to reveal the static and dynamic characteristics of the Islamic FinTech in selected articles into the following areas: the co-occurrence network of authors keywords, countries and institutes, and the word cloud.

*Co-occurrence network of authors keywords*

**Figure 4.**
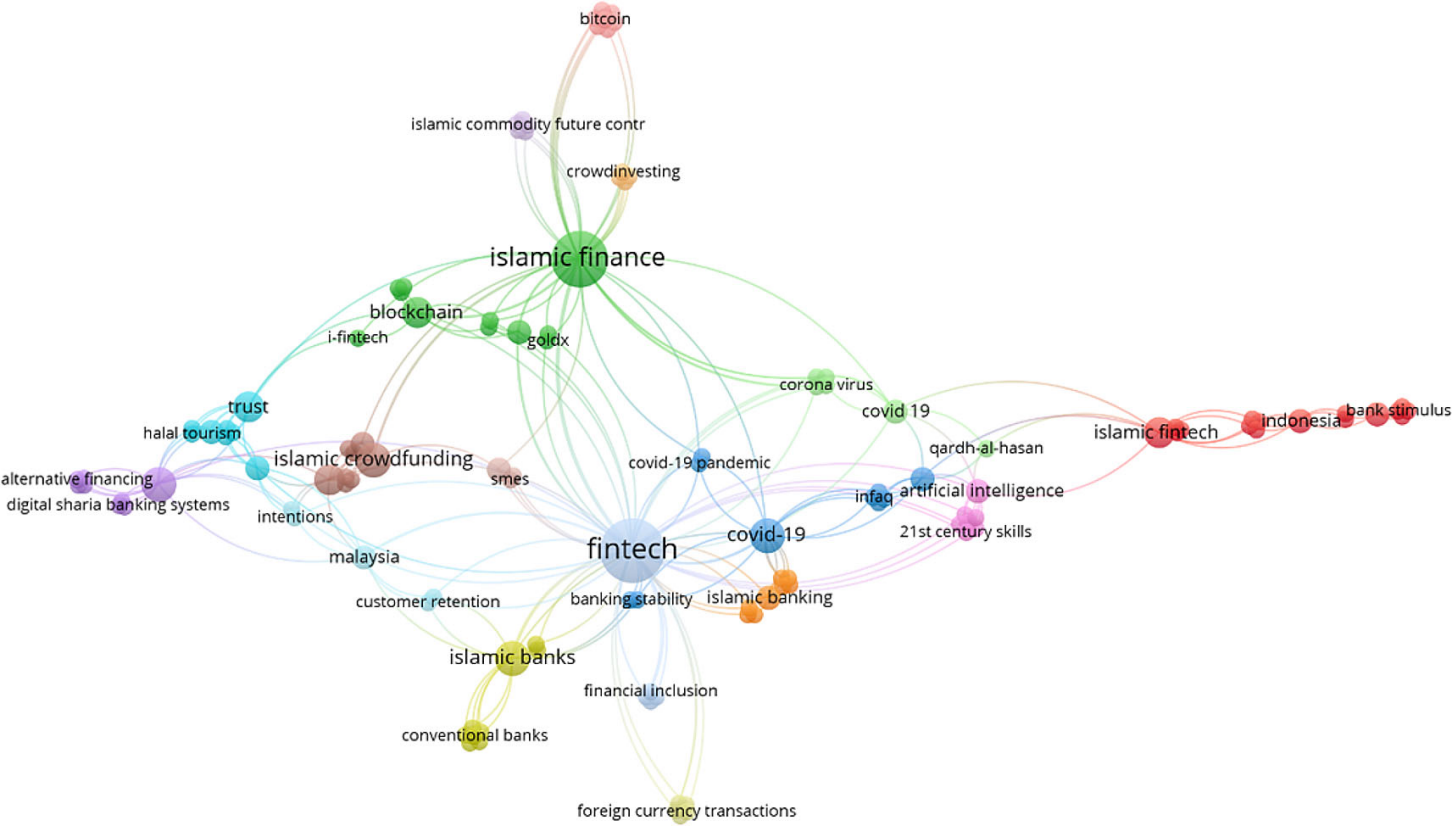
Co-occurrence network of authors keywords.

Figure 4 shows the frequently used keywords in the Islamic FinTech literature from 2016 to 2021 in the selected articles. The most common keywords found are “FinTech”, “Islamic Finance”, “Covid 19 or Coronavirus or Covid-19 pandemic or Covid-19”, and “Crowdfunding”.

*The collaboration network of countries*

**Figure 5.**
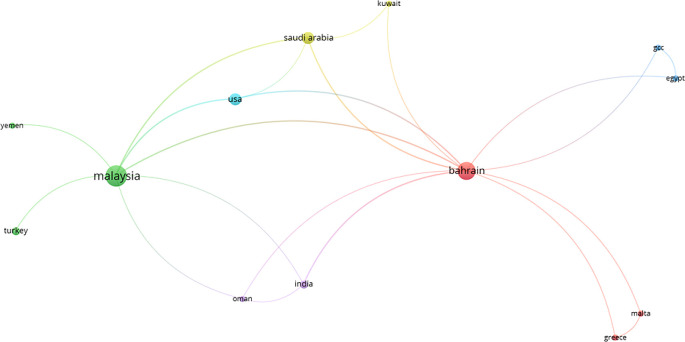
The collaboration network of countries.

Figure 5 shows the collaboration network between different countries regarding Islamic FinTech. Also, it shows that Malaysia and Bahrain are more active in Islamic FinTech, and there is cooperation between Malaysia and Bahrain with other countries in this field. However, the efforts of other countries remain minimal and unpretentious in the field of Islamic FinTech.

*The collaboration network of authors*

**Figure 6.**
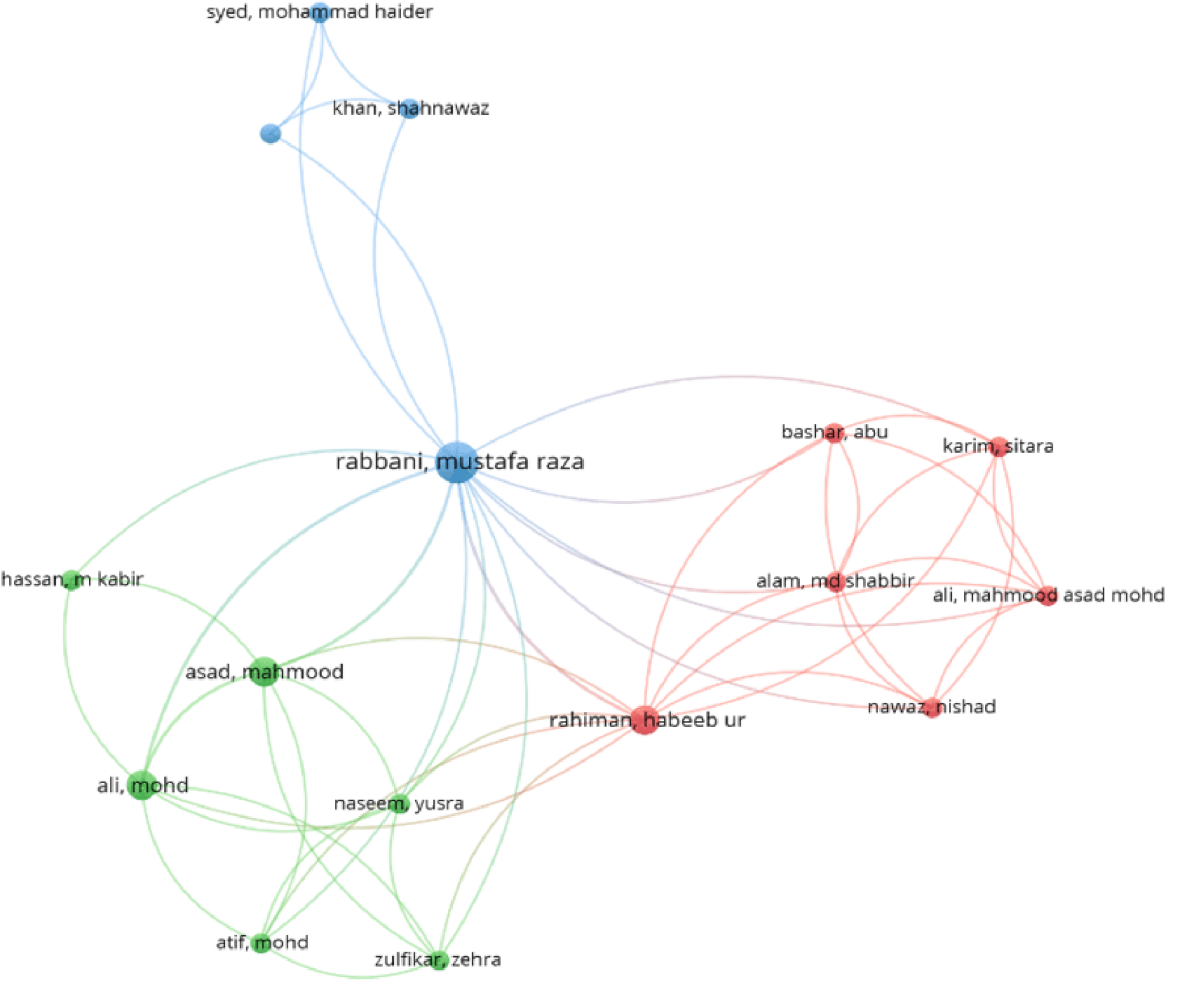
The collaboration network of authors.

Figure 6 highlights the collaboration among authors of the Islamic FinTech articles. Mustafa Raza Rabbani, Habeeb Ur Rahiman, Mohammed Ali and Mahmood Asad are the top authors in the Islamic FinTech publications.

*The collaboration network of institutes*

**Figure 7.**
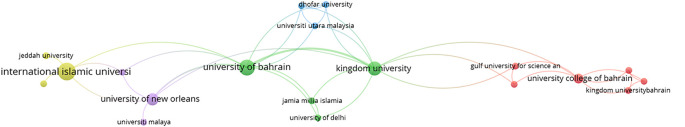
The collaboration network of institutes.

Figure 7 shows the most active universities and institutes in Islamic FinTech. The University of Bahrain, the International Islamic University, and Kingdom University are among the top institutions in the Islamic FinTech publications from other universities.

*Word cloud*

Using keyword occurrence in NVivo software we found that the word “Islamic” in the selected articles was repeated 3339 times. Then, the term “technology” was repeated 1580 times, while “fintech” was found 1561 times. The word “crowdfunding” was found 1319 times in the selected articles.

### Other analyses

The Journal of Islamic Marketing is ahead of other journals in the number of research publications in Islamic FinTech. The number of its publications reached six research papers, followed by the International Journal of Islamic and Middle Eastern Finance and Management with five articles. Both journals are published by Emerald Group Publishing Ltd., United Kingdom ( Table 4).

**Table 4.** Top six journals in Islamic FinTech.

Journal	Publisher	Country	H-Index	Rank	PY-Start	Total articles published	Papers
Journal of Islamic Marketing	Emerald Group Publishing Ltd.	United Kingdom	39	Q2	2010	6	(Hendratmi *et al*., 2021), ( Darmansyah, Fianto, Hendratmi, & Aziz, 2020), ( Usman, Mulia, Chairy, & Widowati, 2020), ( Berakon I., Wibowo M.G., Nurdany A., & Aji H.M., 2021), ( Ishak *et al*., 2021) and (Berakon I., Aji H.M., & Hafizi M.R., 2021)
International Journal of Islamic and Middle Eastern Finance and Management	Emerald Group Publishing Ltd.	United Kingdom	29	Q2	2010	5	( Nastiti & Kasri, 2019), ( Selim, 2021), ( Altwijry, Hassan, & Selim, 2021), ( Banna, Hassan, Ahmad, & Alam, 2021), ( Ascarya, 2021)
Qualitative Research in Financial Markets	Emerald Group Publishing Ltd.	United Kingdom	17	Q3	2010	3	( Rosavina, Rahadi, Kitri, Nuraeni, & Mayangsari, 2019), ( Chong, 2021), ( Ishak & Rahman, 2021),
International Journal of Economics and Business Administration	International Strategic Management Association	Greece	13	Q2	2018	2	( Syed, Khan, Rabbani, & Thalassinos, 2020), ( Rabbani, Khan, & Thalassinos, 2020)
Journal of Islamic Accounting and Business Research	Emerald Group Publishing Ltd.	United Kingdom	20	Q3	2010	2	( Busari & Aminu, 2022), ( Shaikh S. A., 2021)
Journal of Open Innovation: Technology, Market, and Complexity	MDPI Multidisciplinary Digital Publishing Institute	Switzerland	22	Q2	2015	2	( Rabbani, *et al*., 2021a), ( Rabbani, *et al*., 2021b)

FinTech (financial technology).

## Discussion

This research shows interest in Islamic FinTech research, which has attracted researchers' passion, supporting this vital industry. It should be noted that the top trend in the Islamic FinTech is mainly related to the regulation business model (horizontal business model) associated with Islamic FinTech ( Gundogdu, 2016; Baber H., 2019a; Nastiti & Kasri, 2019; Muryanto, Kharisma, & Ciptorukmi Nugraheni, 2021; Darmansyah, Fianto, Hendratmi, & Aziz, 2020; Altwijry, Hassan, & Selim, 2021; Hasnan, 2021). However, the number of studies in this business model does not exceed seven research papers. It is also remarkable that the interest in Islamic FinTech increased with the emergence of the COVID-19 pandemic (M., Rabbani, Asad, & Ali, 2020; Syed, Khan, Rabbani, & Thalassinos, 2020; Banna, Hassan, Ahmad, & Alam, 2021; Ascarya, 2021; Rabbani *et al*., 2021a; Rabbani, Khan, & Tjalassions, 2020). It is natural to search for solutions for Islamic banking in light of the continuation of this epidemic until writing this research. Moreover, confirming what we have found through this research, a lot of vertical business model research was in Islamic crowdfunding ( Abdullah & Umar, 2017; Saiti, Musito, & Yucel, 2018; Rahman, Mohd Thas Thaker, & Duasa, 2020; Hendratmi, Ryandono, & Sukmaningrum, 2019; Hasnan, 2021; Syed, Khan, Rabbani, & Thalassinos, 2020; Salim, Kassim, & Thaker, 2021; Ishak, Kamaruddin, & Aderemi, 2021; Ascarya, 2021; Ishak & Rahman, 2021). The crowdfunding vertical business model could be a solution for individuals and small and medium-sized companies affected by the Coronavirus.

Since Islamic FinTech is in the process of production, testing, and the exchange of results and experiences, the challenges of regulation for this industry are considered among the most significant challenges that have been raised and discussed through the selected research ( Abdullah & Umar, 2017; Nastiti & Kasri, 2019; Jailani, Al-Aaidroos, Mukhtar, Abu Bakar, & Ismail, 2020; Muryanto, Kharisma, & Ciptorukmi Nugraheni, 2021; Selim, 2021; Darmansyah, Fianto, Hendratmi, & Aziz, 2020; Rabbani, Khan, & Tjalassions, 2020; Busari & Aminu, 2022; Kirchner, 2020; Altwijry, Hassan, & Selim, 2021). Islamic cryptocurrency regulations, engaging the Islamic FinTech in money creation, compliance with Shariah law, eliminating “riba” via Islamic FinTech, and Sukuk offerings are issues discussed in the regulation management challenge by different authors.

On the other hand, the adoption and acceptance of various groups of people for Islamic FinTech were crucial challenges posed. Their factors were analyzed in the articles selected for this research. Customer management challenge ( Baber H., 2019a; Rosavina, Rahadi, Kitri, Nuraeni, & Mayangsari, 2019; Usman, Mulia, Chairy, & Widowati, 2020; Shaikh *et al*., 2020; Berakon, Wibowo, Nurdany, & Aji, 2021) that includes customer retention and satisfaction through Islamic FinTech products were discussed in the selected articles. Furthermore, how consumer trust plays a vital role in adopting and accepting the Islamic FinTech using integrity, ability, and benevolence was another issue negotiated in the selected articles. Moreover, some of the papers chosen discussed the impact of halal knowledge on customer satisfaction and retention through Islamic FinTech.

The emergence of the COVID-19 pandemic at the beginning of 2020 posed another challenge ( Rabbani *et al*., 2021a; Syed, Khan, Rabbani, & Thalassinos, 2020; Banna, Hassan, Ahmad, & Alam, 2021; Ascarya, 2021) to Islamic FinTech. It was discussed how Islamic FinTech could find solutions to this pandemic and help those affected. It was found that it may constitute alternatives and solutions to financial and sustainability problems for small and medium companies and individuals through an alternative Islamic acceptable to the Muslim community and complies with the laws of Sharia.

By investigating the keywords of the research selected for this research through bibliometric analysis, we found that the term “FinTech” was repeated more than other keywords, followed by “Islamic finance”, then “COVID-19” or related, and finally “Crowdfunding”. This emphasizes the unity of context for the selected research.

We found through this research that there is a cooperation between countries and researchers, primarily from Malaysia and Bahrain. This explains the extent of interest in Islamic FinTech and the development and growing volume of Islamic banking in these countries. Furthermore, this study found that Rabbani, Habeeb, Mohammed Ali and Asad are the top authors in Islamic FinTech. These authors are active and cooperate with other authors to produce articles in Islamic FinTech. Moreover, the authors' top three universities commonly affiliated with more Islamic FinTech articles: International Islamic University, University of Bahrain, and Kingdom University.

The COVID-19 pandemic posed a tremendous and profound challenge to banks in general. The COVID-19 pandemic has damaged the global economy and negatively affected individuals and companies, especially small and medium companies, causing the closure of many of them and the layoff of a large number of workers from their jobs around the world. Furthermore, the complete and intermittent closure of banks in some countries, especially those witnessing a revival of the Islamic banking market, affected it in terms of its daily operation and the ability of customers to communicate with it in these circumstances. Thus, FinTech has become one of the leading solutions adopted by banks. On the other hand, Islamic FinTech has also been a way to support the Islamic banking sector. The issues related to the COVID-19 pandemic was the center of selected articles, including clarifying the role of Islamic FinTech in combating the effects of the pandemic, studying the impact of the pandemic on the number of transactions of the Islamic banking sector, what new technology, services and strategies have resulted from the COVID-19 pandemic, and how to take advantage of FinTech and innovations to overcome the effects of the pandemic.

This research has several limitations, such as it relies on articles recorded only in the Scopus database. Furthermore, it included studies that were written in English only. It also adopted the challenges from studies by Lee and Shin (2018) and Li and Xu (2021) and did not consider the others.

### Practical implications

Overall, the availability of literature review in Islamic FinTech is still tiny, modest, and straightforward if measured by the volume of research in various horizontal and vertical business models of conventional banking. This study has several practical implications. It reveals gaps in the literature reviews on Islamic FinTech. The articles that have been selected for this research follow a slow and singular approach. This trend involves crowdfunding business models, lending platforms, and the regulation challenges of these business models. There are no clear studies regarding technologies such as AI and their applications in Islamic FinTech that meet the laws of Sharia. Also, studies are not available regarding big data and how to use it to conform with Sharia laws and then apply it in Islamic FinTech.

Furthermore, there is a lack of research that studies the deviations of technologies such as the blockchain and their impact on the conformity of Sharia in Islamic FinTech. This study will contribute to directing researchers towards these gaps to fill them in the service of the progress of Islamic FinTech. In addition, it will give a clear picture of the most critical challenges and recent issues facing the Islamic FinTech sector for financial institutions. COVID-19 brought a lot of challenges and issues; therefore, this study highlights the most critical issues facing Islamic FinTech, and scholars can further investigate the impact of this challenge on Islamic FinTech.

Based on the statistics presented in the introduction related to Islamic banking and FinTech, researchers need to study the various issues, challenges and problems facing the Islamic FinTech sector. Further studies are required focusing on horizontal-technology business models such as blockchain, big data and open sources/APIs business models that constitute a robust supportive base for Islamic banks and finance, who want to compete with conventional banks and FinTech startup companies in Islamic and Arab countries. Furthermore, according to Dawood, Liew and Lau (2021), FinTech in the future will be driven by the preferences of the new generation who are adopting digital services through mobile technology. Hence, more research needs to be conducted in the Islamic FinTech context covering the Islamic FinTech ecosystem's elements such as regulation management, risk management, customer management and others.

## Data availability

### Underlying data

All data underlying the results are available as part of the article and no additional source data are required.

### Reporting guidelines

Dryad: PRISMA checklist for “Business Trends & Challenges in Islamic FinTech: A Systematic Literature Review”. https://doi.org/10.5061/dryad.bzkh189bq ( Dawood *et al*., 2022).

Data are available under the terms of the Creative Commons Zero “No rights reserved” data waiver (CC0 1.0 Public domain dedication).

## Footnotes

^1^
Arabic and Islamic narration refer to games of chance where there are always two parties: a winner and a loser.

^2^
Arabic and Islamic narration include the games of chance, which includes the generation of income randomly and by luck, and it is considered immoral.

^3^
Arabic and Islamic narration mean the measurement of uncertainty, and Islam sees it as difficult to measure and ensure that a person not be deceived due to unclear contract terms.

^4^
Arabic and Islamic narration (asking the borrower to return more money on the original loan amount).

^5^
Arabic and Islamic narration for sharing (forming a partnership).

^6^
Arabic and Islamic narration for cost-plus financing.

^7^
Retrieved from https://www.iedcdubai.ae/wp-content/uploads/2019/02/SGIE-Report-2018-19_Eng_1540649428.pdf (accessed on 11.12.2021).

^8^
Retrieved from https://omannews.gov.om/Arabic_NewsDescription/ArtMID/437/ArticleID/43187/Home (accessed on 11.12.2021).

^9^
Retrieved from https://assets.kpmg/content/dam/kpmg/xx/pdf/2021/08/pulse-of-fintech-h1.pdf (accessed on 11.12.2021).

^10^
Retrieved from https://blog.cfte.education/the-fintech-unicorn-hubs-of-2021-where-do-the-largest-fintechs-hail-from/ (accessed on 23.12.2021).

^11^
Retrieved from https://www2.deloitte.com/content/dam/Deloitte/in/Documents/financial-services/in-fs-open-banking-report-noexp.pdf (accessed on 20.12.2021).

^12^
Data extracted on 12.12.2021 ( https://www.scopus.com/home.uri).

^13^
Retrieved from https://guides.lib.lsu.edu/c.php?g=376856&p=2550574 (accessed on 23.12.2021).

^14^
Retrieved from https://authorservices.taylorandfrancis.com/publishing-your-research/writing-your-paper/different-types-of-research-articles/ (accessed on 23.12.2021).

^15^

https://www.scimagojr.com/.

^16^
Alternative free software (Gate: https://gate.ac.uk/ and RQDA: http://rqda.r-forge.r-project.org/).

## References

[ref1] Abdeldayem MM Dulaimi SH al Dulaimi FH al : A qualitative approach to evaluate the reconciliation of GOLDX and OneGram in Islamic Finance. *J. Econ. Bus.* 2021;39(1):113–134. 10.18045/zbefri.2021.1.113

[ref2] Abdullah S Umar AO : Towards a sharī’ah compliant equity-based crowdfunding for the halal industry in Malaysia. *International Journal of Business and Society.* 2017;18(1):223–240.

[ref3] Ahmi A Mohamad R : Bibliometric Analysis of Global Scientific Literature on Web Accessibilityl. *International Journal of Recent Technology and Engineering.* 2019:250–258.

[ref4] Almulla D Aljughaiman AA : Does financial technology matter? Evidence from an alternative banking system. *Cogent Economics & Finance.* 2021;9. 10.1080/23322039.2021.1934978

[ref5] Altwijry OI Hassan MK Selim M : Developing a Shari’ah based FinTech money creation free [SFMCF] model for Islamic banking. *The International Journal of Islamic and Middle Eastern Finance and Management.* 2021. 10.1108/IMEFM-05-2021-0189

[ref6] Ascarya A : The role of Islamic social finance during Covid-19 pandemic in Indonesia’s economic recovery. *The International Journal of Islamic and Middle Eastern Finance and Management.* 2021. 10.1108/IMEFM-07-2020-0351

[ref7] Auwal Adam Sa’ad Ahmad K Saleh AO : P2P Islamic FinTech Investment Innovation. *Journal of Islamic Thought and Civilization.* 2019.

[ref8] Baber H : FinTech, Crowdfunding and Customer Retention in Islamic Banks. *Vision: The Journal of Business Perspective.* 2019a;24(3):260–268. 10.1177/0972262919869765

[ref9] Baber H : Relevance of e-SERVQUAL for determining the quality of FinTech services. *International Journal of Electronic Finance, Inderscience Enterprises Ltd.* 2019b;9(4):257–267. 10.1504/IJEF.2019.104070

[ref10] Banna H Hassan MK Ahmad R : Islamic banking stability amidst the COVID-19 pandemic: the role of digital financial inclusion. *The International Journal of Islamic and Middle Eastern Finance and Management.* 2021. 10.1108/IMEFM-08-2020-0389

[ref11] Belanche D Casaló LV Flavián C : Artificial Intelligence in FinTech: understanding robo-advisors adoption among customers. *Ind. Manag. Data Syst.* 2019;119(7):1411–1430. 10.1108/imds-08-2018-0368

[ref12] Berakon I Wibowo MG Nurdany A : An expansion of the technology acceptance model applied to the halal tourism sector. *Journal of Islamic Marketing.* 2021. 10.1108/JIMA-03-2021-0064

[ref14] Bican PM Brem A : Digital Business Model, Digital Transformation, Digital Entrepreneurship: Is There A Sustainable “Digital”?. *Sustainability.* 2020;12(13):5239. 10.3390/su12135239

[ref15] Busari SA Aminu SO : Application of blockchain information technology in Ṣukūk trade. *Journal of Islamic Accounting and Business Research.* 2022;13(1):1–15. 10.1108/JIABR-10-2019-0197

[ref16] Casino F Dasaklis TK Patsakis C : A systematic literature review of blockchain-based applications: Current status, classification and open issues. *Telematics Inform.* 2019;36:55–81. 10.1016/j.tele.2018.11.006

[ref17] Chong FL : Enhancing trust through digital Islamic finance and blockchain technology. *Qualitative Research in Financial Markets.* 2021;13(3):328–341. 10.1108/QRFM-05-2020-0076

[ref18] Darmansyah D Fianto BA Hendratmi A : Factors determining behavioral intentions to use Islamic financial technology. *Journal of Islamic Marketing.* 2020;12(4):794–812. 10.1108/jima-12-2019-0252

[ref19] Dawood HM Liew CY Lau TC : Mobile perceived trust mediation on the intention and adoption of FinTech innovations using mobile technology: A systematic literature review. *F1000Res.* 2021;10. 10.12688/f1000research.74656.1 PMC9021664 35464181

[ref20] Dawood H : Business Trends & Challenges in Islamic FinTech: A Systematic Literature Review, Dryad, Dataset. 2022. 10.5061/dryad.bzkh189bq PMC9253660 35844816

[ref21] Deng L Lv Y Liu Y : Impact of Fintech on Bank Risk-Taking: Evidence from China. *Risks.* 2021;9(5):99. 10.3390/risks9050099

[ref22] Fernandez-Vazquez S Rosillo R Fuente D de la : Blockchain in FinTech: A Mapping Study. *Sustainability.* 2019;11(22):6366. 10.3390/su11226366

[ref23] Gundogdu AS : Islamic electronic trading platform on organized exchange. *Borsa Istanbul Rev.* 2016;16(4):249–255. 10.1016/j.bir.2016.06.002

[ref24] Hasnan B : Examining the Intentions to Use Crowdfunding Platform - An Extended Technology Acceptance Model. *International Journal of Services, Economics and Management.* 2021.

[ref25] Hendratmi A Ryandono MH Sukmaningrum PS : Developing Islamic crowdfunding website platform for startup companies in Indonesia. *Journal of Islamic Marketing.* 2019;11(5):1041–1053. 10.1108/jima-02-2019-0022

[ref26] Hendratmi Ryandono Sukmaningrum : Developing Islamic crowdfunding website platform for startup companies in Indonesia. *Journal of Islamic Marketing.* 2020;11(5):1041–1053. 10.1108/JIMA-02-2019-0022

[ref27] Hommel K Bican PM : Digital Entrepreneurship in Finance: Fintechs and Funding Decision Criteria. *Sustainability.* 2020;12(19):8035. 10.3390/su12198035

[ref28] Imerman MB Fabozzi FJ : Cashing in on innovation: a taxonomy of FinTech. *J. Asset Manag.* 2020;21(3):167–177. 10.1057/s41260-020-00163-4

[ref29] Ishak Kamaruddin Aderemi : Mudharabah based crowdfunding as an alternative source of funding book publications in Malaysia. *Journal of Islamic Marketing.* 2021. 10.1108/JIMA-05-2020-0147

[ref30] Ishak MI Rahman MH : Equity-based Islamic crowdfunding in Malaysia: a potential application for mudharabah. *Qualitative Research in Financial Markets.* 2021;13(2):183–198. 10.1108/QRFM-03-2020-0024

[ref31] Jailani N Al-Aaidroos M Mukhtar M : Mapping E-Auction Sharia Compliant Requirements to User Interface Design. *International Journal on Advanced Science.* 2020;10(3):1058–1065. 10.18517/ijaseit.10.3.10266

[ref32] Kirchner IF : Are Cryptocurrencies ḥalāl? On the Sharia-Compliancy of Blockchain-Based Fintech. *Islamic Law and Society.* 2020; (1-2):76–112. 10.1163/15685195-BJA10005

[ref33] Kou G Olgu Akdeniz Z Dinçer H : Fintech investments in European banks: a hybrid IT2 fuzzy multidimensional decision-making approach. *Financial Innovation.* 2021;7(1):1. 10.1186/s40854-021-00256-y PMC8138114 35024283

[ref34] Lee I Shin Y : Fintech: Ecosystem, business models, investment decisions, and challenges. *Bus. Horiz.* 2018;61(1):35–46. 10.1016/j.bushor.2017.09.003

[ref35] Lenz R : Peer-to-Peer lending – opportunities and risks. *European Journal of Risk Regulation.* 2016, December;1–21.

[ref36] Li B Xu Z : Insights into financial technology (FinTech): a bibliometric and visual study. *Financial Innovation.* 2021;7(1):28–69. 10.1186/s40854-021-00285-7 35024290 PMC8492101

[ref37] Hassan MK Rabbani MR Asad M : Challenges for the Islamic Finance and banking in post COVID era and the role of Fintech. *Journal of Economic Cooperation and Development.* 2020.

[ref38] Muryanto YT Kharisma DB Ciptorukmi Nugraheni AS : Prospects and challenges of Islamic fintech in Indonesia: a legal viewpoint. *International Journal of Law and Management.* 2021.

[ref39] Najaf K Subramaniam RK Atayah OF : Understanding the implications of FinTech Peer-to-Peer (P2P) lending during the COVID-19 pandemic. *Journal of Sustainable Finance & Investment.* 2021:1–16.

[ref40] Nasir A Shaukat K Khan KI : What is Core and What Future Holds for Blockchain Technologies and Cryptocurrencies: A Bibliometric Analysis. *IEEE Access.* 2021;9:989–1004. 10.1109/access.2020.3046931

[ref41] Nastiti ND Kasri RA : The role of banking regulation in the development of Islamic banking financing in Indonesia. *The International Journal of Islamic and Middle Eastern Finance and Management.* 2019;12(5):643–662. 10.1108/IMEFM-10-2018-0365

[ref42] Nguyen VA Nguyen TPT : An Integrated Model of CSR Perception and TAM on Intention to Adopt Mobile Banking. *The Journal of Asian Finance, Economics and Business.* 2020;7(12):1073–1087. 10.13106/jafeb.2020.vol7.no12.1073

[ref43] Nour Aldeen K Ratih IS Sari Pertiwi R : Cash waqf from the millennials' perspective: a case of Indonesia. *ISRA International Journal of Islamic Finance.* 2021. 10.1108/IJIF-10-2020-0223

[ref44] Ooi K-B Tan GW-H : Mobile technology acceptance model: An investigation using mobile users to explore smartphone credit card. *Expert Syst. Appl.* 2016;59:33–46. 10.1016/j.eswa.2016.04.015

[ref45] Ozili PK : Impact of digital finance on financial inclusion and stability. *Borsa Istanbul Rev.* 2018;18(4):329–340. 10.1016/j.bir.2017.12.003

[ref46] Pranay G Mandy TT : *FinTech, The new DNA of Financial Services.* Boston/Berlin: Walter de Gruyter Inc;2019.

[ref47] Pustokhina IV Pustokhin DA Mohanty SN : Artificial intelligence assisted Internet of Things based financial crisis prediction in FinTech environment. *Ann. Oper. Res.* 2021;1–21. 10.1007/s10479-021-04311-w

[ref48] Rabbani MR Khan S Thalassinos EI : FinTech, Blockchain and Islamic Finance: An Extensive Literature Review. *European Research Studies Journal.* 2020;348–367.

[ref49] Rabbani MR Bashar A Nawaz N : Exploring the Role of Islamic Fintech in Combating the Aftershocks of COVID-19: The Open Social Innovation of the Islamic Financial System. *Journal of Open Innovation: Technology, Market, and Complexity.* 2021a;7(2):136. 10.3390/joitmc7020136

[ref50] Rabbani RM Asad Mohd MA Rahiman HU : The Response of Islamic Financial Service to the COVID-19 Pandemic: The Open Social Innovation of the Financial System. *Journal of Open Innovation: Technology, Market, and Complexity.* 2021b;7(1):85. 10.3390/joitmc7010085

[ref51] Rahman MP Mohd Thas Thaker MA Duasa J : Developing a Sharīʿah-compliant equity-based crowdfunding framework for entrepreneurship development in Malaysia. *ISRA International Journal of Islamic Finance.* 2020;12(2):239–252. 10.1108/IJIF-07-2018-0085

[ref52] Reiners L : *FinTech Law and Policy.* Coppell, TX, USA: Independently published;2018.

[ref53] Rosavina M Rahadi RA Kitri ML : P2P lending adoption by SMEs in Indonesia. *Qualitative Research in Financial Markets.* 2019;11(2):260–279. 10.1108/QRFM-09-2018-0103

[ref54] Ryu Ko KS : Sustainable Development of FinTech: Focused on Uncertainty and Perceived Quality Issues. *Sustainability.* 2020;12(18):7669–7686. 10.3390/su12187669

[ref55] Ryu H-S : What makes users willing or hesitant to use Fintech?: the moderating effect of user type. *Industrial Management & Data.* 2018;118(3):541–569. 10.1108/IMDS-07-2017-0325

[ref56] Saiti B Musito MH Yucel E : Islamic Crowdfunding: Fundamentals, Developments and Challenges. *The Islamic Quarterly.* 2018;62(3):469.

[ref57] Salim M Kassim S Thaker MM : Factors influencing the acceptance of Islamic crowdfunding in Malaysia: A study of youth entrepreneurs. *Pakistan Journal of Commerce and Social Sciences.* 2021:443–475.

[ref58] Selim M : The effects of eliminating Riba in foreign currency transactions by introducing global FinTech network. *The International Journal of Islamic and Middle Eastern Finance and Management.* 2021;14(3):506–523. 10.1108/IMEFM-01-2020-0035

[ref59] Shaikh IM Qureshi MA Noordin K : Acceptance of Islamic financial technology (FinTech) banking services by Malaysian users: an extension of technology acceptance model. *Foresight.* 2020;22(3):367–383. 10.1108/fs-12-2019-0105

[ref60] Shaikh SA : Using Fintech in scaling up Islamic microfinance. *Journal of Islamic Accounting and Business Research.* 2021;12(2):186–203. 10.1108/JIABR-10-2019-0198

[ref61] Syed MH Khan S Rabbani MR : An Artificial Intelligence and NLP based Islamic FinTech Model Combining Zakat and Qardh-Al-Hasan for Countering the Adverse Impact of COVID 19 on SMEs and Individuals. *Int. J. Econ. Bus. Adm.* 2020:351–364.

[ref62] To A Trinh THM Corona CG : Understanding behavioral intention to use mobile wallets in vietnam: Extending the tam model with trust and enjoyment. *Cogent Business & Management.* 2021;8(1):1–14. 10.1080/23311975.2021.1891661

[ref63] Todorof M : Shariah-compliant FinTech in the banking industry. *ERA Forum.* 2018;19(1):1–17. 10.1007/s12027-018-0505-8

[ref64] Usman H Mulia D Chairy C : Integrating trust, religiosity and image into technology acceptance model: the case of the Islamic philanthropy in Indonesia. *Journal of Islamic Marketing.* 2020.

[ref65] Vejačka M Štofa T : Influence of security and trust on electronic banking adoption in Slovakia. *E+M Ekonomie a Management.* 2017;20(4):135–150. 10.15240/tul/001/2017-4-010

[ref66] Visconti RM : FinTech Valuation. 2020.

[ref67] Wang JS : Exploring biometric identification in FinTech applications based on the modified TAM. *Financial Innovation.* 2021;7(1):1–24. 10.1186/s40854-021-00260-2

[ref68] Zouari G Abdelhedi M : Customer satisfaction in the digital era: evidence from Islamic banking. *Journal of Innovation and Entrepreneurship.* 2021;10(1):1. 10.1186/s13731-021-00151-x

