# Supplementary material for: Business trends & challenges in Islamic FinTech: A systematic literature review
Source: F1000Res. 2022 Mar 18;11:329. [Version 1] doi: 10.12688/f1000research.109400.1 (PMC9253660; doi:10.12688/f1000research.109400.1)
Supplement: Peer review history

# Peer review: Reviewer response for version 1

**DOI:** 10.5256/f1000research.120895.r139921

**Published:** 2022-07-04

**Authors:** Maha Shehadeh

**Affiliations:** 1 Department of Accounting, Faculty of Business, Middle East University, Amman, Jordan

Thank you for inviting us to conduct a review of this high-quality article paper entitled "Business trends and challenges in Islamic Fintech: a systematic literature review".

 We think this paper is excellent and it is a good idea. We really liked how this paper highlighted the challenges and recent trends in Islamic financial technology. We have seen how the authors built their analysis based on very solid theoretical foundation which makes the article an amazing work. The method and the software employed is the most appropriate one in the literature.

  In fact, We just have some minor comments:

1. Figure 2. "FinTech Challenges to Business Models". It is better to put this figure on page (9) that discusses the challenges of FinTech, rather than in the introduction.
2. This manuscript needs to indicate the importance of the research, so that the reader feels the importance of the scientific addition provided by this manuscript.
3. It is preferable to emphasize in the body of the research that financial technology is a technical issue and has many benefits for Islamic financial institutions, and there is nothing to prevent its use in Islamic financial institutions as long as it does not conflict with the provisions and principles of Islamic Sharia.
4. We have a minor note about the 'regulation management challenge'. In our opinion, money laundering, privacy and security concerns should be placed with the challenge of risks associated with FinTech. As for 'the specific rules and regulations for innovation', it is already considered one of the most important challenges of financial technology; the issue of the lack of legislation regulating financial technology and its failure to keep pace with digital transformation is one of the most important challenges that hinder financial and banking institutions in their path towards digital transformation.
5. Some abbreviations are not mentioned in full in the content of the paper, like: SLR which could be not clear enough for the readers who are out of the field.
6. We recommend that the authors provide more details about the journals (data) employed in their analysis (Research Data and Sample Construction) and how they reached the final sample number and the sources they used: journals, databases, the journal ranking etc.
7. Adding a table summarising the data employed divided into themes and sub-themes based on: the contexts, the data of publication, theories employed, or the fields will be much easier for the reader to understand.

Once again many thanks, We hope our comments help the authors and We will be pleased to assist in the future,

 Regards,

 Maha & Esraa

Are the rationale for, and objectives of, the Systematic Review clearly stated?

Yes

Is the statistical analysis and its interpretation appropriate?

Yes

Are sufficient details of the methods and analysis provided to allow replication by others?

Yes

Are the conclusions drawn adequately supported by the results presented in the review?

Yes

Reviewer Expertise:

(Maha):  FinTech, Cryptocurrencies, Islamic Digital Finance, Blockchain, Financial Markets, Islamic Finance, digital transformation.  (Esraa): Auditing, International standards, IFRS Accounting.

I confirm that I have read this submission and believe that I have an appropriate level of expertise to confirm that it is of an acceptable scientific standard.

# Peer review: Reviewer response for version 1

**DOI:** 10.5256/f1000research.120895.r127920

**Published:** 2022-04-07

**Authors:** Dr Naz Farah

**Affiliations:** 1 Department of Education, University Management and Technology, Lahore, Pakistan

Thank you very much for giving me the opportunity to review the manuscript "Business trends & challenges in Islamic FinTech: A systematic literature review". I have reviewed this paper thoroughly. The study offers interesting insights. However, the study needs to address a few comments given below:

1. The introduction needs to show the importance of this study, contribution to the literature, and the importance of Islamic Fintech and major policy recommendations.
2. In the conclusion section, future research avenues and limitations need to be elaborated
3. I found some grammatical and typo errors in the manuscript.

Further to these points, I believe that the addition of a flow chart depicting both horizontal and vertical aspects would improve the article by providing an overview of the content available at a glance.

Are the rationale for, and objectives of, the Systematic Review clearly stated?

Yes

Is the statistical analysis and its interpretation appropriate?

Yes

Are sufficient details of the methods and analysis provided to allow replication by others?

Yes

Are the conclusions drawn adequately supported by the results presented in the review?

Yes

Reviewer Expertise:

Finance

I confirm that I have read this submission and believe that I have an appropriate level of expertise to confirm that it is of an acceptable scientific standard.
